# New York Odontological Society

**Published:** 1895-03

**Authors:** 

**Affiliations:** New York Odontological Society


### Reports of Society Meetings.

### NEW YORK ODONTOLOGICAL SOCIETY.

A REGULAR meeting of the New York Odontological Society was held on Tuesday evening, November 20, 1894, at the New York Academy of Medicine, No. 17 West Forty-third Street, New York City, with the President, Dr. Brockway, in the chair.

The secretary read the minutes of the previous meeting, which were approved.

### INCIDENTS OF OFFICE PRACTICE AND CASUAL COMMUNICATIONS.

*Dr. Davenport.*—Within the last week I have been so pleased over the accomplishment of something which I have not often done that I decided to speak of it here. A lady who has been for some years wearing a full upper denture recently consulted me because the plate would not keep its place while eating or talking. While the plate fitted accurately and had good suction when it was made, it has recently—the mouth being very soft—become exceedingly loose, and it seemed imperative at first to make a new plate, very much against the wish of both the lady and myself, for the proper arrangement of the teeth in her case takes a great deal of time. As it happened, she possessed a full upper rubber plate, which she had not worn for a number of years, and the teeth upon that plate were entirely satisfactory to her in appearance, but of course the plate did not fit. I suggested experimenting with the old plate, to see if I could not so change it that it would answer her purpose without much expenditure of time. A plaster impression of the mouth was taken, the band of the plate was cut down almost to the teeth, and the entire centre cut out, so that nothing was left but sufficient rubber to hold the teeth in place. This being placed upon the plaster cast and waxed up as usual, was next tried in the mouth and slightly changed to obtain the correct articulation, after which it was finished in the usual way. The result was satisfactory, and the whole thing was accomplished with a very small expenditure of time. I thought it worth while to bring this to the attention of the Society, because very often, with people over forty years of age, duplicate plates will accumulate, and often it may be possible to make those changes easy for patient and operator, and still bridge the patient over a number of years.

*Dr. Hodson.*—I do not want to steal Dr. Davenport's thunder, but many years ago, in my student days, Dr. S. B. Palmer used regularly to do that sort of thing for his patients, so making the permanent plate out of the temporary one. He had a little circular saw, with which he could cut out the centre of the plate, and often made the permanent plate in that way.

To go back to our incidents of office practice, I would be glad of some suggestion, if any gentleman has any new one to apply to the always difficult and unsatisfactory condition of a plate carrying the lower back teeth, the natural front ones from cuspid to cuspid being *in situ*. In this particular case—the most discouraging one that my brother (whose specialty that department is) has ever seen —it has been impossible by any means which his ingenuity could devise to hold the plate firmly and comfortably in position. We have even considered the possible propriety of sacrificing the cuspids for the purpose of holding posts for a bridge; but hope to find something else or some other way to accomplish the result desired.

*Dr. Littig.*—Are the teeth entirely out back of the cuspids?

*Dr. Hodson.*—Yes. I have been hoping that somebody might have something new to suggest. I have never seen one that was nearly successful where the clasp around the front gum was used, while these cuspids are of such shape that it is practically impossible to get any clasping of sufficient value to hold the plate.

*Dr. Littig.*—Why not put a cap on the cuspids?

*Dr. Hodson.*—That could be done, of course, but would be very unsightly, as, from their position and the fact of their being live teeth and too sensitive to cut down much, the caps would enlarge them seriously for the front of the mouth, and either the clasps or sleevings of sufficient strength to hold such a plate under strong mastication coming outside of this would be inadmissible.

*Dr. Jackson.*—I would suggest that the doctor make a slight space, by wedging between the cuspid and the lateral incisor on each side of the mouth, and extend springs of clasps from the plate on the palatal side of the teeth through the spaces, having the plate fitted well to the distal side of them, and letting it extend somewhat towards the front. In that way he will be able to retain the plate firmly.

*Dr. Littig.*—I would state that you cannot make a partial lower plate remain satisfactorily in the mouth where there is not sufficient elevation at the ramus to prevent its sliding back, unless you clasp the teeth in front.

*Dr. Hodson.*—The ridge is nothing but a thread on one side, and is as tender and delicate as possible, so that the cast has to be built up with wax along this knife-like edge, to keep the pressure of the heavy plate upon it from being painful.

Dr. Huntington then read a paper on contour.

### DISCUSSION.

*Dr. Davenport.*—We are all apt to feel that when some one agrees with us, he is a pretty good sort of a fellow. It has been my contention for years that it mattered less what material was chosen for a filling than it did what form the filling was caused to assume. I have even found that in certain mouths where soft teeth were to be treated, that the much-condemned phosphates, if properly and snugly contoured, would make very durable approximal fillings, which in my opinion is more a proof of the value of contouring than of the phosphates. I congratulate Dr. Huntington upon his paper, for I feel that we cannot too often refer to this matter or too forcibly impress upon the young members of the profession who are coming forward that the majority of the dentists of the present day believe the contour filling to be the proper filling for the patient's comfort and the preservation of teeth.

*Dr. Howe.*—I very heartily approve of what Dr. Huntington has advocated so well in his paper. I congratulate him on being able to present the idea that he wished to convey in so impressive a way. I would say, however, that we always expect the essayist to inform us rather than to ask us conundrums, and I wish very much that he had told us, before he concluded his paper, how he fills pin-head cavities in approximal surfaces of molars.

*Dr. Davenport.*—I did not intend to pose as an advocate of zinc-phosphate, desiring only to emphasize my preference for contour fillings even of the poorest materials over flat fillings of more durable materials. I still assert, however, that in many cases zinc-phosphate contour fillings, made of Smith's adamantine cement, Poulson's cement, or the so-called Harvard cement, have given me great satisfaction.

*Dr. Hodson.*—Do you use anything else in your cervical margins ?

*Dr. Davenport.*—It is my usual custom to use at least a half-matrix for such fillings, and often I pack a thin layer of some dense gutta-percha at the cervical wall.

*Dr. Sailer.*—I would like to emphasize the point about the pin-head cavity between the bicuspids and the molars. While I agree with Dr. Davenport in endorsing the general tenor of the paper, I also agree with Dr. Howe as to the essayist not giving us the benefit of the new ideas which occurred to him. That question has been raised, but not answered. I find that in the majority of cases you can fill pin-head cavities between bicuspids and molars with the point which we all have heard Dr. Dwinelle and Dr. Lord speak about, and tin. I find the most difficult thing about them is the forming of an undercut which will retain the tin. If you can form the undercut, you can fill with tin every time. I hope I have answered the question to Dr. Howe's satisfaction. It is an important question which occurs to every dentist.

*Dr. Jarvie.*—For the sake of bringing out a little discussion, I would state how I fill such cavities. For instance, if the decay is between the first and second molars, and there is a pin-head cavity in the approximal surface of each tooth, I will cut through from the grinding surface of the first molar into the cavity in that molar, and use the space I gain in that way to fill on the approximal surface of the second molar, making a compound cavity in the first molar and a simple cavity in the second.

*Dr. Sailer.*—Would Dr. Jarvie do the same with the bicuspids?

*Dr. Jarvie.*—That is my rule in all approximating cavities of that size between first and second bicuspids, second bicuspids and first molars, or between first and second molars. That is my *rule*, but I do not say I make no exceptions. I cannot fill such cavities satisfactorily unless I cut into one of them from the grinding surface.

*The President.*—Do you see any objection to that method, Dr. Sailer?

*Dr. Sailer.*—I do, because if there is sufficient tooth substance left on the grinding surface of a bicuspid, it should be saved, for as soon as it is lost, we know it is liable to split apart. By a little extra patience on the part of the operator and the patient, you can get the teeth far enough separated to operate on without cutting away any portion of the grinding surface.

Dr. Perry then read his paper entitled "Moderation in Practice and in Statement."

(For Dr. Perry's paper, see page 133.)

DISCUSSION.

*Dr. Jarvie.*—The paper is of such a character that it is almost impossible to discuss it; it is above criticism. It has gone into the merits of moderation both in statement and in practice so thoroughly and so well that I have nothing to say except in commendation. I think Dr. Perry has given us a treat to-night, one that we have all enjoyed thoroughly.

*Dr. Howe.*—I not only approve in emphatic though moderate terms of Dr. Perry's position, but I think what he has said is called for. If the mass of dentists will give heed to what has been said in this paper, they will be greatly benefited, and dentistry as an art and as a science will be greatly advanced. I wish especially to commend Dr. Perry's criticism, in the way he has put it, of the abuse of bridge-work and of gold crowns. The exhibition of gold crowns, as described, and as we all have had them thrust upon our notice, is one of the disgraces of the period.

*Dr. Davenport.*—Some of the best novels of the day are based upon historical events, and Dr. Perry's delightful paper, to which we have listened this evening, seems to be based upon the life of the Father of our Country, who was "First in war, first in peace, and first in the hearts of his countrymen."

I would like to suggest a modification of that and apply it to the essayist,—"First in theory, first in practice, and first in the hearts of his fellow-dentists."

Adjourned.

John J. Hart, D.D.S.,
*Editor New York Odontological Society.*

